# Outcomes following biosimilar TNF inhibitor use for inflammatory-mediated immune disorders in pregnancy

**DOI:** 10.1177/1753495X211028779

**Published:** 2021-09-02

**Authors:** R Scott, H Parker, S Mccartney, P Harrow, D Williams, I Giles

**Affiliations:** 1 Department of Obstetrics, University College London Hospital NHS Foundation Trust, London, UK; 2 Department of Gastroenterology, University College London Hospital NHS Foundation Trust, London, UK; 3 The Institute for Women’s Health, University College London, London, UK; 4 Department for Rheumatology, University College London Hospital NHS Foundation Trust, London, UK; 5 Centre for Rheumatology, University College London, University College London Hospitals NHS Foundation Trust, London, UK

**Keywords:** Biosimilar, pregnancy, inflammatory, rheumatology, gastroenterology

## Abstract

**Background:**

Biosimilar tumour necrosis factor inhibitors (TNFi) are increasingly used to treat inflammatory immune-mediated disorders as they cost less than the originator biologic drug. More women are therefore becoming pregnant on biosimilar TNFi. This is the first paper to explore the safety and efficacy of biosimilar therapies in pregnancy.

**Methods:**

A retrospective review of clinical data reviewed pregnancy outcomes and inflammatory disease activity in 18 pregnancies where the mother was using a biosimilar TNFi at conception.

**Results:**

Biosimilar therapy was not associated with congenital abnormalities, preterm birth or other adverse pregnancy outcomes. Stopping biosimilar TNFi in pregnancy was associated with childbirth at an earlier gestation, as well as a flare of inflammatory disease in pregnancy or post-partum.

**Conclusions:**

Women and clinicians should feel confident in using biosimilar TNFi in early pregnancy, and continuing them through pregnancy to prevent flares in late pregnancy or the early post-partum.

## Introduction

Biological disease modifying anti-rheumatic drugs (bDMARDs) are complex molecular entities such as antibodies. They target specific inflammatory and immunological molecules such as tumour necrosis factor (TNF). These agents effectively turn off specific aspects of the inflammatory and immune system. They are currently used to treat disorders as disparate as rheumatoid arthritis, psoriasis and inflammatory bowel disease.

Over the last 20 years there has been a shift to rapid and aggressive treatment of inflammatory-mediated immune disorders (IMIDs), with bDMARDS being used early in the disease course. These drugs significantly reduce symptoms, prevent long-term damage and improve quality of life,^1^ though they are associated with an increased risk of infections due to their effects on the immune system.^2^

As many IMIDs affect women of child-bearing age, increasing numbers of women with these conditions are considering pregnancy, and becoming pregnant, with disease controlled by biological therapies.^3^

However, many biologic therapies are very expensive. Biosimilars therapies have been developed to provide a more affordable alternative to the originator biologic drug. Biosimilars are defined as ‘similar to biotherapeutic products of assured quality, safety, and efficacy that have been licensed based on a full licensing dossier’.^4^ Because biological originator drugs are produced from living cells or organisms, inevitable variations exist between different biological systems and manufacturing processes, meaning that the biosimilars will be highly similar but not identical to the originator drug.^5^ Using biosimilars rather than originator biologic drugs in biologic naïve women, and switching from the originator biologic DMARDs to the equivalent biosimilar, saves significant amounts of money. Consequently, biosimilars are increasingly used in women of child-bearing age.

There is, however, little data on the use of biosimilars in pregnancy, with regard to their safety or efficacy. No trials of biosimilars in pregnancy have been published, and current advice is based on the use of original biologic DMARD in pregnancy without confirmation of true safety equivalence of the biosimilar in pregnancy.

We describe a cohort of 18 women with IMIDs treated with TNF inhibitor (TNFi) biosimilar drugs in pregnancy. The maternal characteristics, pregnancy course and outcomes, and disease progression throughout pregnancy and the post-partum period are described to evaluate any adverse outcomes that may be considered in pregnancy counselling for these drugs.

## Method

A retrospective service evaluation was undertaken. Women were identified through cross-referencing a database of women referred to obstetric medicine clinics at a single tertiary maternity centre, with a list of women receiving adalimumab, etanercept and infliximab biosimilar therapies. The database was reviewed from January 2019 until September 2020 to capture all women prescribed TNFi biosimilars in that period. The choice of TNFi was decided by the referring clinician in conjunction with the woman during routine clinical care, and for infliximab, etanercept and adalimumab, the hospital policy was for prescription of the biosimilar in place of the originator drug. The electronic health records of these women were then reviewed. All women had been diagnosed with IMIDs prior to pregnancy, and were established on the biosimilar therapy prior to conception.

Disease control was assessed from the electronic health records. Physician’s global assessment (PGA) measures are global measures of inflammatory disease activity which include information about patient symptoms, clinical examination, radiological and laboratory measures. PGA measures are reliable and simple scales for activity in IMIDs.^6,7^ Where available from the clinical notes, the authors documented the PGA for the patient’s disease activity. If not available, the authors reviewed the clinical notes of the women, and ascribed a PGA measure of baseline and ongoing disease activity based on all available evidence to provide a uniform score of disease activity across the different IMID in place of disease-specific measures (such as the Bath Ankylosing Spondylitis Disease Activity Index (BASDAI), Manual Muscle Testing-8 Score (MMT8) and Disease Activity Score-28 (DAS) score). The PGA incorporated patient-reported symptoms as well as laboratory and clinical data. PGA options were ‘well-controlled’, ‘mild activity’, ‘moderate activity’ and ‘severe activity’. Flare severity in pregnancy was also defined using a PGA with ‘mild’, ‘moderate’ or ‘severe’ as options.

All statistical analyses were performed using GraphPad Prism8. Mean, median and ranges were documented depending on the distribution of continuous data. Comparisons were made using *t*-tests, Mann–Whitney U tests and χ^2^ tests as appropriate.

## Results

We identified 18 women who were exposed to a TNFi biosimilar drug in pregnancy (Table 1). No other biosimilar therapies were used. All women were being treated with a biosimilar at conception. Average length of biologic drug therapy prior to conception was five years (1–12 years).

**Table 1.** Baseline characteristics of women who use biosimilar therapies in pregnancy.

Age in years (mean, range)	34 (22–41)
Ethnicity (n)	
Black African/Afro-Caribbean	3 (17%)
Caucasian	13 (72%)
South Asian	2 (11%)
Nulliparity (n, %)	9 (50%)
Underlying inflammatory disorder	
Ankylosing spondylitis	4
Seropositive rheumatoid arthritis	3
Seronegative arthritis	1
Psoriatic arthritis	1
Crohn’s disease	6
Ulcerative colitis	1
Enteropathic arthritis and Crohn’s disease	1
Juvenile dermatomyositis and Crohn’s disease	1
Years since diagnosis (mean, range)	10 (3–18)
Years of treatment with biologic DMARD (mean, range)	5 (1–12)

Seven women continued their biosimilar throughout pregnancy, while 11 women (11/18) stopped their biosimilar therapy in pregnancy. The biosimilar was stopped in the first trimester in 2/18 women, 8/18 women in the second trimester and 1 woman in the third trimester. Those who stopped their biosimilar in the second and third trimesters did so in collaboration with the clinician managing their condition, whereas stopping the biosimilar in the first trimester was patient-led.

Nine women were treated with biosimilar therapy for inflammatory rheumatic diseases (including rheumatoid arthritis, psoriatic arthritis and ankylosing spondylitis); six women had inflammatory bowel disease (Crohn’s disease and ulcerative colitis) and two women had both inflammatory bowel and rheumatic diseases (juvenile dermatomyositis with Crohn’s disease and seronegative enteropathic arthritis). The average time from diagnosis to this pregnancy was 10 years (3–18 years).

Prior to conception, disease activity was well controlled in 15/18 women (Table 1). Nine women (9/18) had a flare of disease within pregnancy or the post-partum. Flares occurred in pregnancy or the early post-partum in 7/11 women who stopped their biosimilar and 2/7 who continued their biosimilar. The flare was mild in five women and moderate in four. The flare was treated with steroids in two women, and biosimilar therapy was restarted in six women. No specific treatment was required in two women.

All 18 pregnancies culminated in live births (Table 2). Average gestation at delivery was 39 weeks (36 weeks and 6 days to 41 weeks and 1 day). One birth was preterm (36 weeks and 6 days) with the woman induced for intrahepatic cholestasis of pregnancy. Average birth weight was 3221 g (±122 g), and the average birth weight centile was 34th (4th–99th).

**Table 2.** Obstetric outcomes of women who continued or stopped their biosimilar DMARD in pregnancy.

	Continued biosimilar (*n* = 7)	Stopped biosimilar (*n* = 11)	
Flare in pregnancy/early post-partum	2	7	*ns*
Gestation at delivery, weeks+days	39^+5^ (38^+5^–41^+1^)	38^+5^ (36^+6^–39^+5^)	*p *=* *0.02
Mode of delivery			
Vaginal delivery	4	3	*ns*
Elective caesarean section	3	7	*ns*
Emergency caesarean section	0	1	*ns*
Birthweight, g (±SEM)	3257 (±141)	3200 (±171)	*ns*
Birthweight centile, mean (range)	31 (5–59)	36 (4–95)	*ns*

Seven women had a vaginal delivery, 10 had an elective caesarean section and one had an emergency caesarean section.

Those who stopped their biosimilar delivered significantly earlier than those who did not (38 weeks and 5 days vs. 39 weeks and 5 days, *p *=* *0.02), even excluding the woman with obstetric cholestasis. There was no significant difference in mean birthweight between the two groups (3.2 ± 0.1kg vs 3.3 ± 0.1kg) or average birthweight centile on the WHO growth chart (31st vs. 36th) (Table 2).

No infants required admission to the neonatal unit, and no congenital abnormalities were found on the Newborn Infant Physical Examination (NIPE), though one baby was described as having a ‘floppy larynx’. A total of 16 women were breastfeeding on discharge from hospital. By three months post-partum, 6/11 women who had stopped their biosimilar therapy in pregnancy had restarted treatment, with a total of 13 women on biosimilar therapy at 3 months post-partum.

## Discussion

To our knowledge, this study is the first published report that directly examines the use of biosimilar agents used to treat rheumatic and gastroenterological inflammatory conditions in pregnancy. One published abstract reports on the use of biosimilar infliximab in pregnancy in 20 women with underlying inflammatory bowel disease.^8^ This abstract reported a cleft palate in one baby, and raised no concerns about the safety of biosimilar TNFi therapies. Though these reports on biosimilars are small, they mirror meta-analyses which show no increased risk of congenital malformations with TNFi originator biologic drugs.^9–11^ The safety of biologic drugs in early pregnancy is also supported by mechanistic studies. Both adalimumab and infliximab are complete IgG1 antibodies, while etanercept is a fusion protein containing a human IgG1 Fc portion bound to the TNF receptor. These drugs all require active transport across the placenta via the FcRn receptors on syncitiotrophoblasts^12,13^ and only small amounts of maternal IgG begin to cross the placenta in early pregnancy.^14^

IgG transport does increase in a linear fashion through each trimester.^13^ TNFi have been detected in neonatal blood at up to 12 months of age, though clearance typically occurs within eight months.^15,16^ Concerns that this process may cause significant immune suppression in babies have not been substantiated clinically, with several studies showing no increased risk of infections in children exposed *in utero* to biological therapy up to one year of age.^11,17–19^ There is, however, one reported case of an infant who died after disseminated Bacille Calmette-Guerin (BCG) infection following vaccination at three months of age. The infant’s mother had received infliximab 10 mg/kg every eight weeks for the treatment of Crohns’ disease throughout pregnancy.^20^

Concern about neonatal immune suppression has led to some international societies recommending that TNFi are stopped during pregnancy. The British Society of Rheumatology (BSR), European League against Rheumatism (EULAR) and American College of Rheumatology recommend preferentially stopping TNFi in the second or third trimesters of pregnancy, though acknowledge they can be continued thorough pregnancy if indicated. The British Society for Gastroenterology and European Crohn’s and Colitis Organisation also recommend discussing the risks and benefits of continuing TNFi medications with pregnant women, though advise that TNFi should continue throughout pregnancy in those with active disease or high risk of relapse. In contrast the American Gastroenterological Association recommends continuing TNFi throughout pregnancy.

Within our cohort, 64% of those women who stopped their biosimilar therapy had a flare in their inflammatory disease in late pregnancy or in the early post-partum period. Stopping TNFi therapy early in pregnancy has been shown to be a risk for flare of IMIDs in pregnancy;^27,28^ our data suggest that the risk of inflammatory disease flare persists even if the biological therapy is continued into the middle of pregnancy before being stopped. Flares of inflammatory disease in pregnancy increase the risk of low birth weight and preterm delivery.^29–31^ From our data, stopping biosimilar TNFi therapies is also a significant risk factor for earlier delivery in women with IMIDs. This clinical observation does not allow us to understand the underlying cause for this association, but it is tempting to postulate that it may be related to an increase in maternal disease activity precipitating delivery.

Our findings demonstrating the safety of the biosimilar TNFi therapies agree with those of multiple other studies of originator TNFi.^9,10^ Therefore, not only should women on biosimilar therapies feel confident to conceive while using these drugs, but it would appear prudent that continuation of biosimilar TNFi therapy throughout pregnancy should be the norm rather than the exception to prevent later disease flares.

Given the small number of cases reported here, all conclusions must be supported by future studies and registry data. However, as the use and range of biosimilars increase, it is important to have some evidence that these drugs can be used with increasing confidence in pregnancy. This information will empower pregnant women on biological therapy to have more informed conversations about the risks and benefits of continuing biosimilar therapies throughout pregnancy.
